# Mutations in UBA3 Confer Resistance to the NEDD8-Activating Enzyme Inhibitor MLN4924 in Human Leukemic Cells

**DOI:** 10.1371/journal.pone.0093530

**Published:** 2014-04-01

**Authors:** G. Wei Xu, Julia I. Toth, Sara R. da Silva, Stacey-Lynn Paiva, Julie L. Lukkarila, Rose Hurren, Neil Maclean, Mahadeo A. Sukhai, Rabindra N. Bhattacharjee, Carolyn A. Goard, Patrick T. Gunning, Sirano Dhe-Paganon, Matthew D. Petroski, Aaron D. Schimmer

**Affiliations:** 1 Ontario Cancer Institute, Princess Margaret Cancer Centre, University Health Network, Toronto, Ontario, Canada; 2 NCI-designated Cancer Center, Sanford-Burnham Medical Research Institute, La Jolla, California, United States of America; 3 Department of Chemistry, University of Toronto Mississauga, Mississauga, Ontario, Canada; 4 HalTech Regional Innovation Centre, Sheridan Institute of Technology and Advanced Learning, Oakville, Ontario, Canada; 5 Division of Nephrology, Children's Hospital Boston, Harvard Medical School, Boston, Massachusetts, United States of America; Ludwig-Maximilians University, Germany

## Abstract

The NEDD8-activating enzyme (NAE) initiates neddylation, the cascade of post-translational NEDD8 conjugation onto target proteins. MLN4924, a selective NAE inhibitor, has displayed preclinical anti-tumor activity in vitro and in vivo, and promising clinical activity has been reported in patients with refractory hematologic malignancies. Here, we sought to understand the mechanisms of resistance to MLN4924. K562 and U937 leukemia cells were exposed over a 6 month period to MLN4924 and populations of resistant cells (R-K562_MLN_, R-U937_MLN_) were selected. R-K562_MLN_ and R-U937_MLN_ cells contain I310N and Y352H mutations in the NAE catalytic subunit UBA3, respectively. Biochemical analyses indicate that these mutations increase the enzyme’s affinity for ATP while decreasing its affinity for NEDD8. These mutations effectively contribute to decreased MLN4924 potency in vitro while providing for sufficient NAE function for leukemia cell survival. Finally, R-K562_MLN_ cells showed cross-resistance to other NAE-selective inhibitors, but remained sensitive to a pan-E1 (activating enzyme) inhibitor. Thus, our work provides insight into mechanisms of MLN4924 resistance to facilitate the development of more effective second-generation NAE inhibitors.

## Introduction

Ubiquitin (Ub) and ubiquitin-like proteins (Ubls), such as neural precursor cell-expressed developmentally downregulated 8 (NEDD8) and small ubiquitin-related modifier (SUMO), are essential mediators of cellular function [1], [2], [3]. Through multi-step enzymatic cascades, Ub and Ubls are conjugated onto target proteins, marking them for various fates such as degradation, translocation, signaling and regulation of transcriptional activity [4], [5], [6], [7]. In the case of NEDD8, the cascade of its conjugation to target proteins (i.e., neddylation) is initiated by the E1 NEDD8-activating enzyme (NAE), which is a heterodimeric molecule consisting of NAEα (also known as amyloid beta precursor protein-binding protein 1, APPBP1) and NAEβ (also known as ubiquitin-like modifier activating enzyme 3, UBA3). In the first step of the cascade, NAE binds ATP and NEDD8 and catalyzes the formation of a NEDD8-AMP intermediate, which binds the adenylation domain of NAE. NEDD8-AMP reacts with the catalytic cysteine in UBA3 during which NEDD8 is transferred to the catalytic cysteine, resulting in a high energy thiolester linkage. NAE then binds ATP and NEDD8 to generate a second NEDD8-AMP, forming a fully-loaded NAE carrying two activated NEDD8 molecules (i.e., one as a thioester and the other as an adenylate) [8], [9], [10]. The thioester-bound NEDD8 is subsequently transferred onto the catalytic cysteine of an E2 NEDD8-conjugating enzyme and finally covalently conjugated to lysine residues of substrate proteins with the help of an E3 NEDD8 ligase.

Mediating cross-talk between Ub and Ubl pathways, neddylation plays a crucial role in the assembly and function of members of the largest family of E3 Ub ligases, the cullin-RING ligases (CRLs). CRLs target a plethora of cellular proteins for ubiquitination and proteasomal degradation, including a number of substrates such as IκBα and p27 that play important roles in cancer progression [11], [12], [13], [14], [15], [16].

Recently, The Takeda Oncology Company: Millennium reported the development of an AMP mimetic, MLN4924, which selectively inhibits NAE [17]. This compound is not a simple substrate-competitive inhibitor; its inhibitory activity is mechanism-based [18]. MLN4924 forms a stable covalent adduct with NEDD8 in the NAE catalytic pocket by reacting with thiolester-linked NEDD8 bound to the enzyme’s catalytic cysteine. Unlike the labile NEDD8-AMP intermediate, the NEDD8-MLN4924 adduct cannot be utilized in subsequent reactions necessary for NAE activity.

Inhibition of NAE by MLN4924 in human cancer cells results in uncontrolled S-phase DNA replication leading to DNA damage and subsequent cell death through apoptosis [17], [19], [20]. MLN4924 shows potent anti-tumor activity in human solid epithelial tumor xenografts [17], and also displays preclinical activity in vitro and in vivo in hematologic malignancies, including leukemia [21], [22], [23]. Currently, this drug is being evaluated in early phase clinical trials in patients with refractory hematologic malignancies including leukemia [24], where it is showing promising clinical efficacy in refractory patients [25]. While still in the early stages of clinical development, the encouraging preclinical and clinical activity of MLN4924 supports investigation into the mechanisms of sensitivity and resistance to this drug [26], [27].

In this report, we describe two previously unreported and uncharacterized novel mutations in the UBA3 gene in two leukemia cell lines with acquired resistance to MLN4924. We demonstrate that these mutations decrease sensitivity of NAE to the drug by changing the biochemical properties of the enzyme without impairing its normal enzymatic function. Interestingly, the MLN4924-resistant cells remain sensitive to a pan-E1 inhibitor known as Compound 1 that is structurally related to MLN4924. Thus, through this study, we have gained important insights into the function of NAE and the basis for the selectivity of NAE inhibitors. In addition, this work will help in the rational development of novel NAE inhibitors to overcome or circumvent resistance to MLN4924.

## Materials and Methods

### Compounds, MLN4924-resistant cell lines and patient samples

MLN4924 and Compound 1 were obtained and prepared as described in “Supporting Information Methods in File S1”. K562 [28] and U937 [29] human leukemia cell lines were obtained as a kind gift from Dr. Kamel-Reid and Dr. Minden (Princess Margaret Cancer Centre, Toronto, ON, Canada), respectively. Both cell lines were authenticated with short tandem repeat (STR) method in September 2011. In addition, cell lines were periodically authenticated by morphologic inspection. K562 and U937 cell lines were cultured in media containing stepwise increasing concentrations of MLN4924 and resistance was periodically assessed by cell viability assay as described in “Supporting Information Methods in File S1”. K562 and U937 cells that grew normally in the presence of 250 nM or 200 nM MLN4924, respectively, were used for further analysis. NEDD8 knockdown was accomplished as detailed in “Supporting Information Methods in File S1”. Mononuclear cells from an AML patient were isolated from the peripheral blood and bone marrow by Histopaque-1077-gradient density centrifugation. The collection and use of human tissue was approved by local ethics review boards (University Health Network, Toronto, ON, Canada; Stanford University, Palo Alto, CA, Protocol no.18329). The AML patient sample was collected at the Stanford Medical Centre with written informed consent and obtained for this study from the Stanford Hematology Division Tissue Bank (https://clinicalinformatics.stanford.edu/projects/hematology.html).

### Immunoblot analyses

Cells were prepared for immunoblot analysis as described in “Supporting Information Methods in File S1”. Immunoblots were performed with anti-NEDD8, anti-ubiquitin (Cell Signaling Technology) and anti-α-tubulin (Calbiochem).

### DNA sequencing

NEDD8 (NM_006156) and UBA3 (NM_003968) cDNAs were amplified by Q-RT-PCR as previously described [30] using the primers listed in Table S1 in File S1. PCR products for NEDD8 (378 bp) and UBA3 (1480 bp) were gel-purified using the QIAEX II Gel Extraction Kit (QIAGEN). Sequencing was performed at the DNA Sequencing Facility of The Hospital for Sick Children (Toronto, ON, Canada).

### Recombinant protein production and kinetic analyses

The production of recombinant human UBA3 (isoform 1) and NEDD8 were performed as described elsewhere [27] and in “Supporting Information Methods in File S1”. UBA3 I310N and UBA3 Y352H were generated using the QuikChange site-directed mutagenesis kit (Agilent). NAE complexes containing UBA3, UBA3 I310N, or UBA3 Y352H were purified by Ni-NTA chromatography [27]. ATP:PPi exchange assays were based on a previously published method [18], [27], [31] and performed essentially as described [27], as detailed in “Supporting Information Methods in File S1”.

## Results

### Selection of K562 leukemia cells resistant to MLN4924

**Figure 1:**
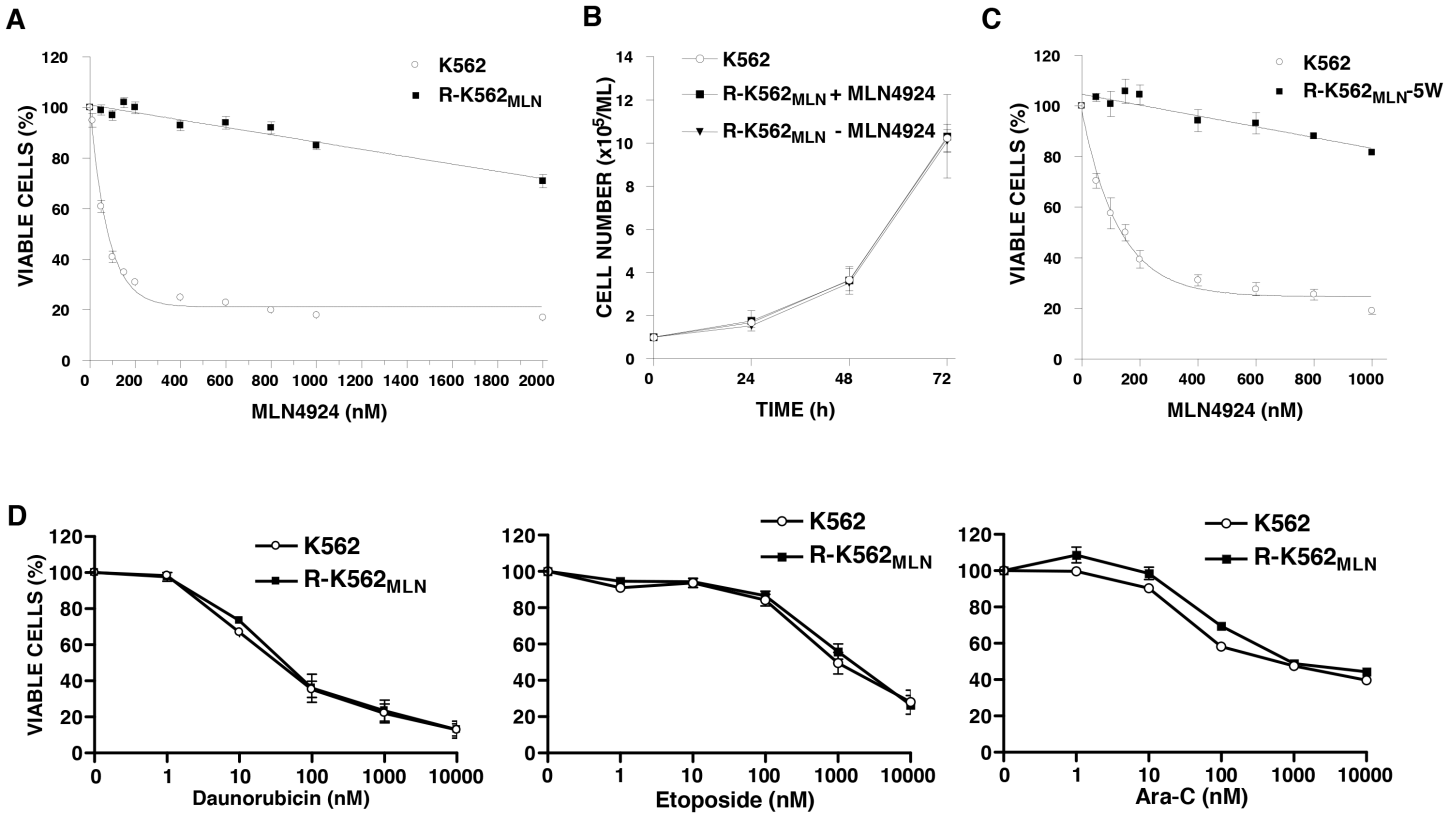
MLN4924-resistant K562 (R-K562_MLN_) leukemia cells show decreased sensitivity to MLN4924, but not all chemotherapeutics. **A**) Cells (5×10^3^ cells/well) were seeded in 96-well plates and treated with increasing concentrations of MLN4924 for 72 hours. After treatment, cell growth and viability were determined by the CellTiter-Glo luminescence assay. Values shown are the mean percentage ± SD of viable cells relative to controls. **B**) Cells (1×10^5^/ml) were plated in 6-well plates. After incubation, cells were stained with trypan blue and counted at the time points indicated. R-K562_MLN_ cells were incubated in the presence or absence of 250 nM MLN4924 as indicated. Values shown represent the means ± SD of viable cells. **C**) Cells (5×10^3^/well) were seeded in 96-well plates and treated with increasing concentrations of MLN4924 for 72 hours. After incubation, cell viability was assessed by the CellTiter Glo assay. Values shown represent the mean percentage ± SD of viable cells relative to vehicle controls. R-K562_MLN_-5W, R-K562_MLN_ cells that were maintained in MLN4924-free medium for 5 weeks. **D**) Cells (5×10^3^/well) were plated in 96-well plates and treated with increasing concentrations of chemotherapeutic drugs as indicated for 72 hours. Cell growth and viability was assessed by the MTS assay. Values shown represent the mean percentage ± SD of viable cells relative to vehicle controls.

The human leukemia cell line K562 was chosen to explore the molecular basis of acquired resistance to MLN4924, as this cell line was sensitive to MLN4924-induced cytotoxicity with EC_50_ values of ∼100 nM (**Figure 1A**). To generate K562 cells resistant to MLN4924, cells were cultured over 6 months in the presence of stepwise increasing concentrations of MLN4924 from 10 to 250 nM. After 6 months, a resistant population of K562 cells was selected that were capable of growth in the presence of 250 nM MLN4924. We termed this subline R-K562_MLN_ and used it for further characterization. To assess the degree of resistance to MLN4924, parental and resistant cells were treated with increasing concentrations of MLN4924. After 72 hours of incubation, cell growth and viability were measured with the CellTiter-Glo luminescence assay. As shown in dose-response curves (**Figure 1A**), R-K562_MLN_ cells were over 40-fold resistant to MLN4924 compared to parental K562 cells. Despite their resistance to MLN4924, R-K562_MLN_ cells proliferated similarly to parental K562 cells (**Figure 1B**). We also evaluated the stability of resistance to MLN4924. R-K562_MLN_ cells were cultured in the absence of MLN4924 for 5 weeks, and sensitivity to MLN4924 was subsequently reassessed. R-K562_MLN_ cells remained resistant to MLN4924 even after withdrawal of the drug (**Figure 1C**).

### MLN4924-resistant K562 leukemia cells remain sensitive to a broad spectrum of chemotherapeutic drugs

In tumour cells, the multidrug resistance (MDR) phenotype is a common mechanism that accounts for the simultaneous emergence of resistance to a variety of anticancer drugs [32], [33]. To exclude the possibility of a generalized MDR phenotype as a mechanism of resistance to MLN4924, we assessed whether R-K562_MLN_ cells demonstrated cross-resistance to other standard chemotherapeutic agents. Parental and resistant cells were treated with increasing concentrations of several chemotherapeutic drugs with distinct mechanisms of action, including daunorubicin, cytarabine, and etoposide. Compared to parental cells, R-K562_MLN_ cells are similarly sensitive to all of the agents tested (**Figure 1D**). Of note, daunorubicin is among the classical substrates of the multidrug efflux transporter P-glycoprotein (P-gp, also known as ABCB1) [33]. The lack of cross-resistance to these drugs in R-K562_MLN_ cells argues against a mechanism of resistance to MLN4924 related to an MDR phenotype. Thus, the mechanism of resistance to MLN4924 appears specific for this agent and not related to generalized multidrug efflux.

### Cullin neddylation is refractory to MLN4924 in K562-resistant cells

**Figure 2:**
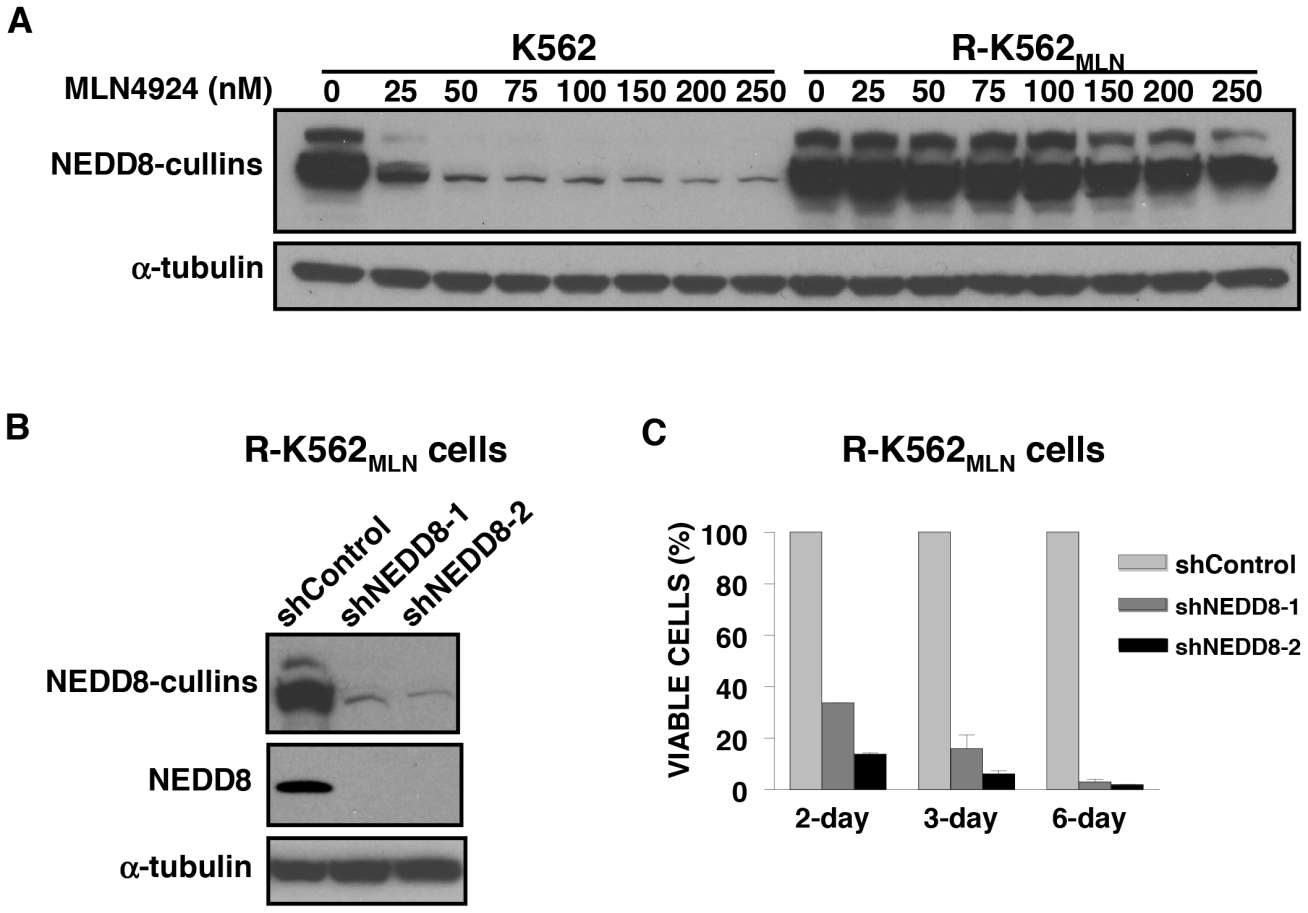
MLN4924-resistant K562 cells show decreased inhibition of cullin neddylation by MLN4924 but remain sensitive to knockdown of NEDD8. **A)** Cells were treated with increasing concentrations of MLN4924 as indicated for 24 hours. After treatment, total cell lysates were prepared and analyzed by SDS-PAGE and immunoblotting using anti-NEDD8 and anti-α-tubulin antibodies to detect NEDD8-cullin complexes and equal protein loading, respectively. **B)** Cells were infected with lentiviral vectors expressing three independent shRNA sequences targeting NEDD8 (shNEDD8) or a control sequence (shControl), and successfully transduced puromycin-resistant populations were selected. Total cell lysates were prepared and analyzed by SDS-PAGE and immunoblotting with antibodies against NEDD8 and α-tubulin. **C)** Cells infected with vectors containing shNEDD8 or control sequences and selected for puromycin resistance were seeded in 6-well plates (1×10^5^ cells/ml). After incubation for 2, 3, and 6 days, cell growth and viability were assessed by trypan blue exclusion. Values represent the mean percentage ± SD of viable cells relative to cells infected with control sequences.

Cullin proteins are the major substrates of the neddylation pathway, and a characteristic feature of NAE activity is the generation of NEDD8-conjugated cullin proteins. We therefore examined the effects of MLN4924 on the levels of these conjugates in parental and resistant cells. Cells were treated for 24 hours with increasing concentrations of MLN4924, and the steady-state abundance of NEDD8-conjugated cullins were assessed by immunoblotting. As shown in Figure 2, the steady state levels of NEDD8-cullins were equivalent in parental and resistant cells. NEDD8-conjugated cullins were diminished in parental cells treated with 25 nM MLN4924 (**Figure 2A**). In contrast, no appreciable reduction in NEDD8-conjugated cullins was observed in R-K562_MLN_ cells after treatment with up to 250 nM MLN4924 (**Figure 2A**).

### NEDD8 is essential for the survival of MLN4924-resistant K562 leukemia cells

To ascertain if the components of the neddylation pathway are intact in MLN4924-resistant cells, we used shRNA to knock down expression of NEDD8 proteins in R-K562_MLN_ cells. Cells were infected with lentiviral vectors encoding NEDD8 shRNA or control sequences. Knockdown of NEDD8 proteins was confirmed by immunoblotting (**Figure 2B**). Knockdown of NEDD8 reduced the steady state abundance of NEDD8-cullins (**Figure 2B**). Moreover, knockdown of NEDD8 was cytotoxic to MLN4924-resistant R-K562_MLN_ cells (**Figure 2C**). These results suggest NEDD8 conjugation remains necessary for the survival of resistant cells.

### MLN4924-resistant K562 cells harbor missense point mutations in UBA3

**Figure 3:**
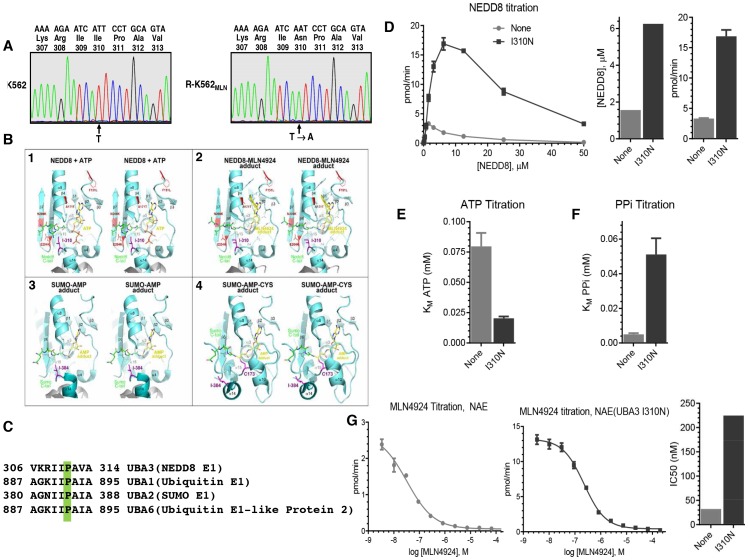
cDNA sequencing reveals missense point mutations in the UBA3 coding region of MLN4924-resistant K562 cells which decrease the sensitivity of NAE to MLN4924 in vitro. **A)** Total cellular RNA was isolated from parental and MLN4924-resistant R-K562_MLN_ cells. UBA3 cDNA spanning its entire coding region was amplified by RT-PCR and then sequenced. The nucleotide sequence from codon 307 to 313 for K562 (*left panel*) and R-K562_MLN_ (*right panel*), three letter amino acid codes, and codon numbers are each shown above the sequence electropherogram tracing. Arrows indicate the position of the second nucleotide of the codon 310 in which the single nucleotide shift (T → A) in R-K562_MLN_ occurred. **B)** Stereoscopic views of NAE and SAE active sites. Enzymes are shown in cyan ribbon format with either NEDD8 or SUMO UBL in green stick format. Secondary structure elements are labelled. **1)** The NAE active site from the PDB = 2NVU structure is shown with NEDD8 and ATP. Also in stick format is I310, which mutated to asparagine renders the enzyme more active than wild type and resistant to MLN4924. For comparison, four other mutations that render resistance are shown in red line format. **2)** The PDB = 3GZN structure is shown with the NEDD8-MLN4924 adduct. Also shown in line format are side chains of residues nearby. **3)** The SUMO-AMP adduct of SAE from PDB = 3KYC shows that a similar isoleucine (I384) is positioned near the diglycine motif of the UBL. **4)** The covalent cysteine intermediate, captured by PDB = 3KYD, shows that I384 must be displaced for reaction to proceed. **C)** Sequence alignment of human UBA3, UBA1, UBA2, and UBA6 was performed, and residues corresponding to UBA3’s I310 are shaded. **D)** NEDD8 (2-fold dilutions from 50 μM) was added to ATP:PPi exchange reactions containing 20 nM NAE or NAE (UBA3 I310N), 1 mM ATP, and 100 μM PPi (supplemented with 50 cpm/pmol [^32^P] PPi). After 30 min at 37°C, radiolabeled ATP generated was measured. Error bars represent SEM (n = 3). Bar graphs indicate the NEDD8 concentration and maximum observed rate for the enzymes. The effects of ATP (**E**) and PPi (**F**) on ATP synthesis were evaluated using either 1.56 μM NEDD8 (NAE) or 6.25 μM NEDD8, determined from (**D**). ATP concentrations tested were 2-fold dilutions from 2 mM with 100 μM PPi. PPi concentrations were 2-fold dilutions from 0.5 mM with 1 mM ATP. **G)** MLN4924 was tested (3-fold dilutions from 200 μM, 2% DMSO final concentration) in reactions containing 20 nM NAE or NAE (UBA3 I310N), 1 mM ATP, 100 μM PPi, and NEDD8. The measured IC_50_ from non-linear regression analyses of triplicate experiments is shown. Error bars represent SEM.

To search for mechanisms of resistance to MLN4924 in the resistant cell line, we sequenced the coding region of NEDD8 and the catalytic subunit of NAE, UBA3, from K562 parental and R-K562_MLN_ cells. No mutations were identified in the NEDD8 gene. However, we identified two missense point mutations in the UBA3 coding region in R-K562_MLN_ cells: an A>T mutation in codon 8 [GAG (Glutamic Acid, E)>GTG (Valine, V)] and a T>A mutation in codon 310 [ATT (Isoleucine, I)>AAT (Asparagine, N)] (**Figure 3A**). Importantly, the homozygous I310N mutation was not present upon sequencing UBA3 cDNA of parental K562 cells. However, the E8V mutation was present in both parental and R-K562_MLN_ cells. Thus, the E8V mutation is likely not responsible for the observed resistance in the R-K562_MLN_ cells. By virtual modeling, I310 is located in the active site of NAE, but is not part of the adenylation pocket. Rather, it lies in the hinge region of the ThiF domain and insertion domain, forming an aliphatic interaction with the penultimate glycine of NEDD8 bound in the adenylation pocket (**Figure 3B**). Of note, sequence alignment showed that the I310 is conserved from mammals to yeast. Moreover, the residue is conserved in other E1 enzymes (**Figure 3C**), further supporting the functional importance of the I310N mutation.

### UBA3 I310N mutation alters NAE biochemical properties to reduce MLN4924 sensitivity

To explore the effects of the I310N mutation on the activity of NAE and its sensitivity to MLN4924, we generated recombinant wild-type and I310N NAE complexes and evaluated these using an ATP:PPi exchange assay [27], [31]. This assay measures the synthesis of radiolabeled ATP from input radiolabeled pyrophosphate (PPi) by NAE in a NEDD8, ATP, and PPi dependent manner, allowing for the quantitative assessment of biochemical properties of the enzyme through titration experiments.

As high concentrations of NEDD8 inhibit ATP synthesis by NAE [31], we first performed NEDD8 titrations to evaluate the effects of the I310N mutation on the reaction rate (**Figure 3D**; raw data shown in **Figure S1A in File S1**). NAE with UBA3 I310N requires higher NEDD8 concentrations than NAE to achieve its maximum rate (6.25 μM and 1.56 μM, respectively), indicating a lower affinity for NEDD8. However, at these NEDD8 concentrations, the maximum rate for the I310N complex is ∼5-fold higher (16.9 pmol/min and 3.3 pmol/min).

Using these NEDD8 concentrations, we next evaluated the effects of ATP (**Figure 3E** and **Figure S1B in File S1**) and PPi (**Figure 3F** and **Figure S1C in File S1**) in the assay. We found that the I310N mutation decreased the K_M_ for ATP of NAE 4-fold (20 μM and 80 μM for the I310N and wild-type complexes, respectively) while the K_M_ for PPi increased ∼10-fold (51.1 μM and 5.0 μM). These data are consistent with the I310N complex having a higher affinity for ATP and lower affinity for PPi than the wild-type enzyme.

To evaluate how these biochemical changes impact the enzyme’s sensitivity to MLN4924, we performed MLN4924 titration experiments (**Figure 3G** and **Figure S1D in File S1**). Consistent with a direct role for the I310N mutation in conferring MLN4924 resistance in cells, this NAE complex was ∼7-fold less sensitive to MLN4924 than wild-type (MLN4924 IC_50_ values are 225 nM and 32 nM, respectively). Together, these biochemical changes in NAE—decreased NEDD8 and PPi affinities, increased ATP affinity, and an increased rate of ATP synthesis— provide a mechanistic basis for acquired MLN4924 resistance found in K562 cells through the UBA3 I310N mutation.

The significant effect of the I310N mutation on kinetics and MLN4924 inhibition is consistent with the fact that this residue is involved in the first and likely rate-limiting step of neddylation, as it forms direct interaction with the diglycine motif of NEDD8 (**Figure 3B**, **panels 1 and 2**).

### U937 leukemia cells become resistant to MLN4924 through a UBA3 mutation distinct from that found in K562 cells

**Figure 4:**
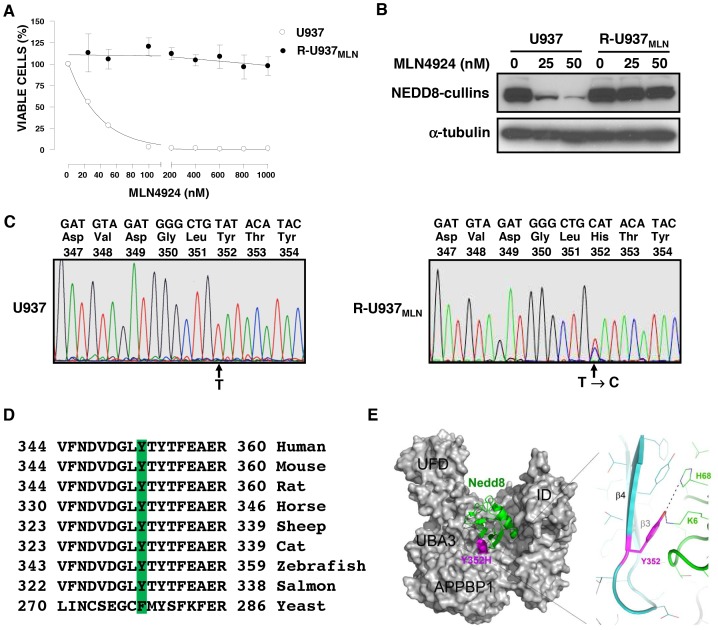
MLN4924-resistant U937 leukemia cells show reduced sensitivity to MLN4924 and are heterozygous for a UBA3 mutation Y352H. **A)** Cells (1×10^4^ cells/well) were seeded in 96-well plates and incubated with increasing concentrations of MLN4924 for 72 hours. After incubation, cell viability was measured by the CellTiter-Glo assay. Values shown are the mean percentage ± SD of viable cells relative to controls. **B)** Cells were treated with increasing concentrations of MLN4924 as indicated for 24 hours followed by isolation of total cellular proteins. Equal amounts of proteins were fractionated by SDS-PAGE and analyzed by immunoblotting with anti-NEDD8 and anti-α-tubulin antibodies. **C)** UBA3 cDNA in parental and MLN4924-resistant R-U937_MLN_ cells was sequenced as described in Figure 4. Shown are the nucleotide sequence from codons 347 to 354 for U937 (left panel) and R-U937_MLN_ cells (right panel), three letter amino acid codes, and codon numbers. Arrows depict the position of the first nucleotide of the codon 352 in which the single nucleotide shift (T → C) in R-U937_MLN_ occurred. **D)** Sequence alignment of UBA3s from different organisms was performed, and residues corresponding to the Y352 are shaded. **E)** The Y352H mutation. To the left the NAE heterodimer surface is shown in grey and its subdomains labelled. Phe352 is shown in magenta. Bound NEDD8 is shown in green ribbon format. On the right, a close-up of the interface between NAE (cyan) and NEDD8 (green) is shown in ribbon format. Shown in line format are residues within proximity.

Having identified a mutation in the UBA3 gene that supports MLN4924 resistance in K562 cells, we asked whether another malignant hematologic cell line employs a similar on-target resistance mechanism. We choose U937 cells as this cell line has been used extensively as a model to study hematological malignancies. We selected a population of U937 leukemia cells resistant to MLN4924 using the method established for K562 cells described above. In cell growth and viability assays, parental U937 cells are sensitive to MLN4924 with an EC_50_ value of 25 nM, while MLN4924-resistant R-U937_MLN_ cells are more than 50-fold less sensitive to MLN4924 (**Figure 4A**). In immunoblot assays, loss of NEDD8-conjugated cullins is observed in parental cells after treatment with 25 nM of MLN4924 for 24 hours (**Figure 4B**), while no significant reduction in the abundance of NEDD8-cullin conjugates occurs in the resistant cells after treatment with 50 nM MLN4924 (**Figure 4B**).

To determine whether UBA3 mutations may be associated with resistance to MLN4924 in R-U937_MLN_, we sequenced the coding region of UBA3 in the parental and R-U937_MLN_ cells. We identified a single point mutation in codon 352 [TAT (Tyrosine, Y) > CAT (Histidine, H)] only in the resistant cells (**Figure 4C**). As there are two overlapping traces representing either the T or C nucleotide at the same position in codon 352 (**Figure 4C**), these cells are heterozygous for the Y352H mutation.

Unlike I310, the Y352 residue is not conserved in other E1 enzymes. However, Y352 is conserved as an aromatic residue from mammals to yeast (**Figure 4D**). In examining the location of Y352 in the NAE structure, this residue is located at the base of the NEDD8 binding pocket, about 20 Å away from I310, where its side chain forms van der Waals interactions with the β1-β2 loop of NEDD8 (**Figure 4E**).

### The Y352H mutation reduces MLN4924 potency on NAE through decreased NEDD8 and increased ATP affinities

**Figure 5:**
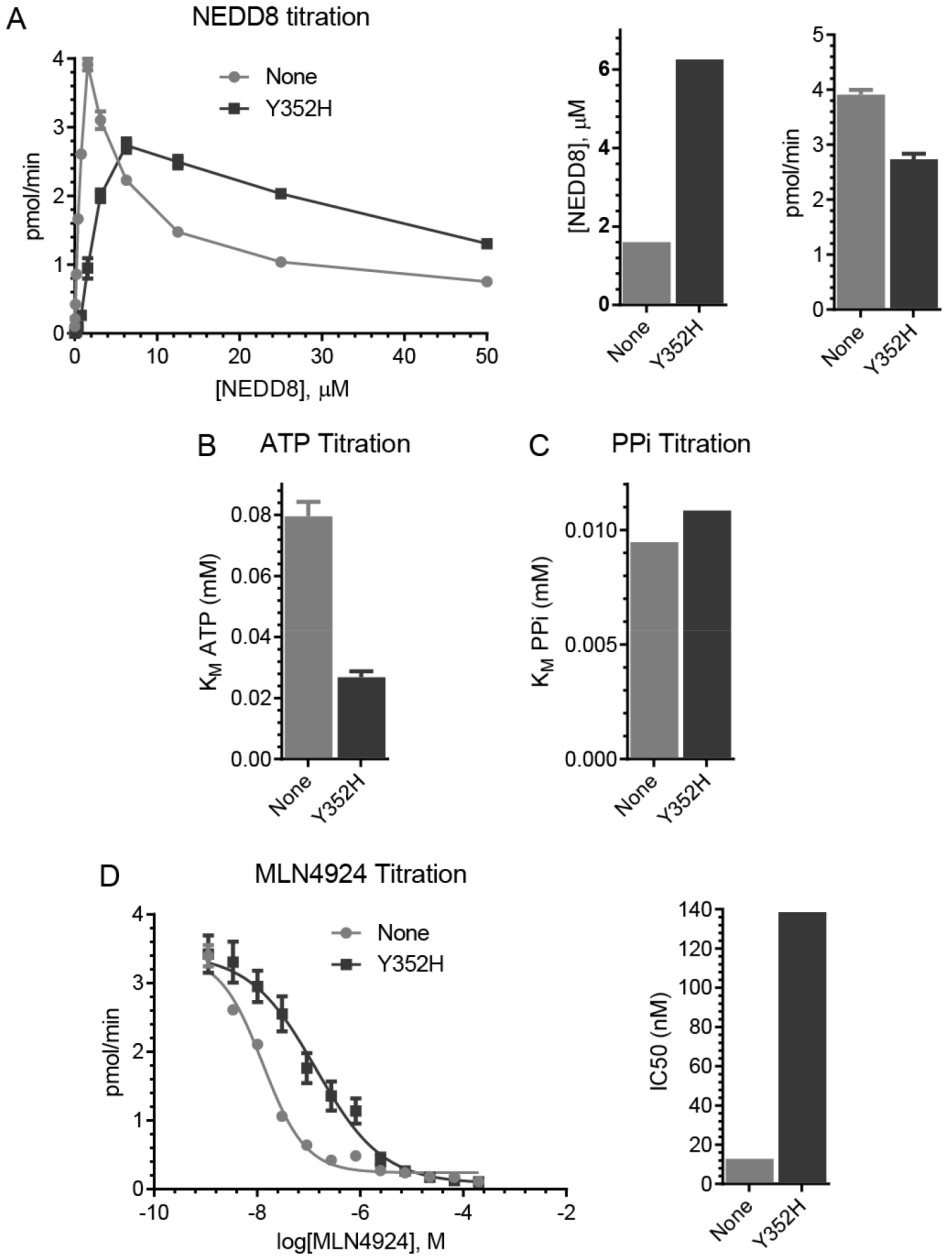
The Y352H mutation is sufficient to decrease the sensitivity of NAE to MLN4924 in vitro. NEDD8 (**A**), ATP (**B**), PPi (**C**), and MLN4924 (**D**) titrations were performed using the ATP:PPi exchange assay to compare ATP synthesis by NAE (UBA3 Y352H) to NAE. Assay conditions were as described in **Figure 3**. Experiments were performed in triplicate with error bars representing SEM.

We compared the rate of ATP synthesis for NAE and NAE (UBA3 Y352H) in the ATP:PPi exchange assay (**Figure 5**). Similar to NAE with the I310N mutation, the Y352H enzyme requires higher NEDD8 concentrations than wild-type (6.25 μM and 1.56 μM) to achieve its maximum rate (**Figure 5A** and **Figure S2A in File S1**). In contrast, however, this rate is ∼1.4-fold lower than wild-type NAE.

In ATP titration experiments (**Figure 5B** and **Figure S2B in File S1**), we found that the Y352H mutation decreases the enzyme’s ATP K_M_ ∼3-fold (27 μM and 80 μM for Y352H and wild-type complexes), similar to what we observed with the complex containing I310N (see **Figure 3E**). However, we found no significant differences in the PPi K_M_ (**Figure 5C** and **Figure S2C in File S1**) for the Y352H complex in comparison to wild-type (10.9 μM and 9.5 μM, respectively), distinct from what we observed with the I310N-containing complex (see **Figure 3F**). In testing this NAE complex with increasing concentrations of MLN4924 (**Figure 5D**), we found that the Y352H mutation confers a ∼10-fold decrease in the sensitivity of NAE to MLN4924 (IC_50_ values 138.5 nM and 13 nM for the Y352H enzyme and wild-type, respectively). These biochemical experiments suggest that the Y352H mutation, like I310N, is sufficient to provide MLN4924 resistance in leukemia cells. Thus, differences in the affinity for PPi may underlie observed differences in ATP synthesis rates between the Y352H and I310N enzyme complexes. Nevertheless, these findings suggest that mutations located in different positions around the NEDD8 binding site of UBA3 cause changes in the catalytic pocket of the enzyme to reduce MLN4924 potency while allowing sufficient NEDD8 system function for leukemia cell survival.

### The pan-E1 inhibitor Compound 1, but not NAE-selective analogues, is active on MLN4924-resistant leukemia cells

Compound 1 is a MLN4924 analogue initially characterized by Millennium Pharmaceuticals (The Takeda Oncology Company) [18]. In contrast with MLN4924, Compound 1 is a pan-E1 inhibitor and is capable of inhibiting a panel of E1 enzymes, including NAE, UBA1 (ubiquitin E1), and UBA2 (SUMO E1) [18], [34]. Through previous structure-activity relationship studies, we generated a library of indane-substituted Compound 1 analogues and identified several that are NAE selective [35].

**Figure 6:**
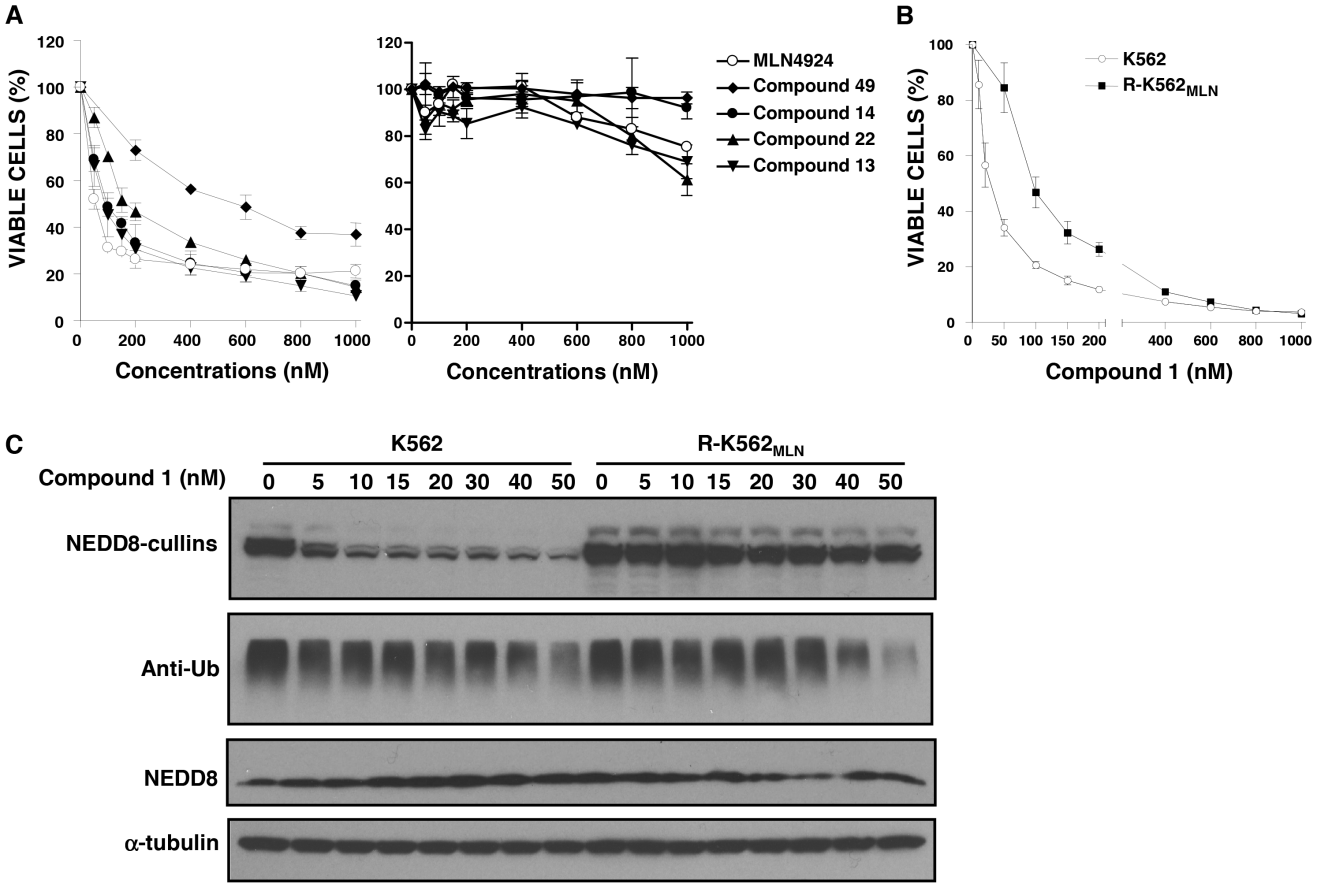
MLN4924-resistant cells are sensitive to the pan-E1 inhibitor Compound 1, but are resistant to NAE-selective Compound 1 analogues. **A)** Cells were seeded in 96-well plates (3×10^3^ cells/well) and treated with increasing concentrations of various selective NAE inhibitors for 72 hours. After treatment, cell viability was assessed by the CellTiter Glo assay. Values shown are the mean percentage ± SD of viable cells relative to vehicle controls. EC_50_ values calculated from the dose-response curves of the parental K562 cells presented here have been presented in a previous publication by Lukkarila *et al* [35]. **B)** Parental K562 and MLN4924-resistant R-K562_MLN_ cells were plated in 96-well plates (5×10^3^ cells/well) and treated with increasing concentrations of compound 1 for 72 hours. After incubation, cell viability was measured by the CellTiter Glo assay. Values shown are the mean percentage ± SD of viable cells relative to controls. **C)** K562 and R-K562_MLN_ cells were treated with increasing concentrations of compound 1 for 24 hours. After treatment, total cellular proteins were analyzed by SDS-PAGE and immunoblotting with anti-NEDD8, anti-ubiquitin (Ub) and anti-α-tubulin antibodies.

We tested these NAE-selective Compound 1 analogues on R-K562_MLN_ cells in viability assays (**Figure 6A**; **Table S2 in File S1**). Although these all have varying potency on the parental K562 cells, which presumably reflect differences in NAE binding mediated by the indane substitutions, the MLN4924-resistant cells are highly cross-resistant to these inhibitors (**Figure 6A** **; Table S2 in File S1**). In immunoblot assays, no significant reduction in the steady state abundance of NEDD8-cullin conjugates was observed in R-K562_MLN_ cells after treatment with these inhibitors for 24 hours at concentrations of 2-fold their respective EC_50_ values in the parental cells, while essentially complete loss of NEDD8-conjugated cullins occurred in parental cells after the treatment (data not shown).

Given that Compound 1 has a broad selectivity profile for other E1s, we reasoned that this molecule may remain cytotoxic to R-K562_MLN_ cells. In comparing the effects of Compound 1 on the parental and R-K562_MLN_ cells in cell viability assays (**Figure 6B**), we found the resistant cells are not refractory to Compound 1 (EC_50_ values of 27 nM and 81 nM respectively). We hypothesize that the 3-fold higher EC_50_ value obtained is due to the inability of Compound 1 to inhibit NAE containing the UBA3 I310N mutation.

In testing this possibility by immunoblotting, extracts from Compound 1 treated cells (**Figure 6C**), we found that parental and R-K562_MLN_ cells have similar changes in ubiquitin conjugates in response to increasing concentrations of Compound 1 while NEDD8-modified cullins in the MLN4924 resistant cells are unaffected. These data are consistent with Compound 1 inhibiting the ubiquitin pathway through UBA1 (and presumably the SUMO E1) in both cell lines, while NAE is only inhibited in the parental K562 cells.

### Leukemic cells in a relapsed AML patient after treatment with MLN4924 do not harbor mutations in the UBA3 gene

Currently, MLN4924 is being evaluated in early phase clinical trials in patients with hematologic malignancies. These early trials are showing promising clinical efficacy in patients with relapsed and refractory AML [25]. To investigate whether UBA3 mutations can explain resistance to MLN4924, we sequenced UBA3 cDNA in a patient with refractory AML who relapsed after an initial response to MLN4924 therapy. An 82-year-old man was diagnosed with therapy-related AML following radiation therapy targeting a localized prostate carcinoma. After progressing on 5-azacitidine, he initiated treatment with MLN4924 at 33 mg/m^2^ intravenously on days 1, 4, 8 and 11 every 21 days in a Phase I trial of the drug (NCT00911066, registered at ClinicalTrials.gov). MLN4924 treatment was well tolerated, and the patient completed 12 cycles of therapy without drug-related adverse events. The patient achieved a partial response following 8 cycles of MLN4924 therapy and a complete remission with incomplete peripheral blood recovery after 10 cycles of therapy. Unfortunately, the patient relapsed after completion of 12 cycles of treatment. Samples of peripheral blood and bone marrow were obtained from the time of diagnosis and relapse, respectively. Mononuclear cells were isolated from the samples and total RNA was extracted. UBA3 cDNA was amplified by Q-RT-PCR and the entire coding region of UBA3 was sequenced and analyzed. No UBA3 mutations were detected at relapse, indicating that relapse of this patient after an initial response to MLN4924 was not caused by a mutation in this gene.

## Discussion

Protein modifications by NEDD8 have important functions promoting cancer cell survival as discovered from pre-clinical studies using the first-in-class NAE inhibitor MLN4924 [17], [20]. In this study, we explored the mechanisms of acquired resistance to MLN4924 using human leukemia cell lines, as early clinical data suggest hematologic malignancies may be the major indication for the drug. We found that both K562 and U937 leukemia cells became resistant to MLN4924 after prolonged exposure to increasing concentrations of the drug. These resistant cells still require NEDD8 for survival, as knockdown of the NEDD8 protein with shRNA decreased viability similar to parental cells.

Inhibition of the neddylation system by MLN4924 requires an active NAE that catalyzes the formation of the inhibitory NEDD8-MLN4924 adduct [26]. This is a unique characteristic of the mechanism-based inhibition of NAE by MLN4924. In our study, we identified two different point mutations in the UBA3 gene of the resistant cell lines that impact NEDD8 binding. We showed that the mutations alter the biochemical properties of NAE. Both increase NAE’s affinity for ATP while requiring higher NEDD8 concentrations for optimal ATP synthesis. Collectively, these features decrease the potency of MLN4924 in vitro and provide a mechanistic basis for the decreased sensitivity of leukemia cells to MLN4924 through these single amino acid mutations.

In addition to the identification of the UBA3 I310N mutation that rendered K562 cells resistant to MLN4924, we also demonstrated that the MLN4924-resistant U937 cells harbor an Y352H mutation in the UBA3 gene. Our results suggest that mutations in the UBA3 gene are a common mechanism for malignant leukemia cells to acquire resistance to MLN4924 in vitro. However, there is a possibility that additional mechanisms are contributing to MLN4924 resistance. While we did not identify any mutations in the coding region of NEDD8, we cannot exclude the possibility that other members of NEDD8 conjugation pathway can also contribute to the development of MLN4924 resistance.

Recently, we and another group have reported the identification of additional MLN4924-induced mutations in the UBA3 gene in several cancer cell lines [26], [27]. Interestingly, mutation at the A171 residue identified in MLN4924 resistant HCT116 colon cancer cell line by both groups was not identified in the two leukemic cell lines used in our current study. It is possible that MLN4924 resistance mutations are tissue specific, especially given that A171 and I310 mutations are located in different regions of UBA3. The A171T mutation is located in the UBA3 nucleotide binding site; other mutations detected by Milhollen *et al* occur at various residues in the NEDD8 binding site of UBA3. Mutations at these sites reduce NEDD8-MLN4924 adduct formation and binding to the NAE [26], [27]. Rather than lying in the nucleotide or NEDD8 binding sites, the I310N mutation identified in our study lies in the hinge region between the ThiF domain and insertion domain of UBA3. The I310 residue forms an aliphatic interaction with the penultimate glycine of NEDD8 bound in the adenylation pocket and may therefore modulate the motion of the insertion domain and the transition of the enzyme from the closed to open state. Mutations in the I310 residue could thereby either produce a physical change in enzyme conformation, or alter the rate at which the insertion domain covers and uncovers the adenylation pocket.

It is interesting to note, that the increase in the NAE’s affinity for ATP in the presence of UBA3 mutations as a resistance mechanism to MLN4924 is reminiscent of the similar drug resistance mechanism in epidermal growth factor receptor (EGFR) mutant. The T790M EGFR mutant has increased affinity for ATP, which is the primary mechanism responsible for the development of resistance to small molecule tyrosine kinase inhibitors (TKIs), such as gefitinib and erlotinib [36]. Since, TKIs compete with ATP in a reversible manner to bind to EGFR, the mechanism of T790M EGFR resistance can be overcome by using second-generation irreversible EGFR inhibitors. Thus, there is a possibility that MLN4924 resistance in UBA3 mutants can be overcome by more potent second-generation NAE inhibitors.

Compound 1, a pan-E1 inhibitor, is capable of inhibiting the enzymatic activity of UBA1, UBA2, and NAE with equal potency [18]. Our studies demonstrated that Compound 1 remained cytotoxic to MLN4924-resistant cells that were also cross-resistant to other selective NAE inhibitors. The cytotoxicity of Compound 1 towards the MLN4924-resistant cells might be explained by its inhibition of other E1 enzymes. Indeed, we demonstrated that Compound 1 diminishes the abundance of ubiquitinated proteins in the resistant cells similar to its effects in parental cells. Thus, pan-E1 inhibitors may overcome resistance to MLN4924 by targeting other E1 enzymes.

It currently remains unknown whether spontaneous or acquired mutations in the UBA3 gene confer resistance to MLN4924 in patients treated with this drug. To our knowledge, we are the first to report the results of sequencing the UBA3 gene in an AML patient who relapsed after achieving remission with MLN4924 treatment. No mutations in UBA3 were detected at either diagnosis or relapse. Thus, additional mechanisms must account for relapse in this patient. Larger numbers of relapsed patients will be needed to determine whether acquired or spontaneous mutations in UBA3 are a clinically relevant cause of relapse.

In summary, we have identified two mutations in UBA3 which were not previously reported or characterized and demonstrated that these mutations confer MLN4924 resistance in leukemia cell lines but do not impair normal function of the NAE enzyme and activity of the neddylation pathway. As MLN4924 continues to be evaluated clinically, our work provides valuable insights that may help detection of MLN4924-resistant malignancies and facilitate the development of second-generation NAE inhibitors to overcome MLN4924 resistance.

## Supporting Information

File S1 **Contains supporting information methods, tables, figures and references.**
(DOC)
