# Correction: Mutations in UBA3 Confer Resistance to the NEDD8-Activating Enzyme Inhibitor MLN4924 in Human Leukemic Cells

**DOI:** 10.1371/journal.pone.0112004

**Published:** 2014-10-22

**Authors:** The PLOS ONE Staff

Dr. Bruno Medeiros is not included in the author byline. He should be listed as the eleventh author and affiliated with Division of Hematology, Stanford University School of Medicine, Stanford, CA, USA. The contributions of this author are as follows: Conceived and designed the experiments, performed the experiments, and analyzed the data.
