# Supplementary material for: Mutations in UBA3 Confer Resistance to the NEDD8-Activating Enzyme Inhibitor MLN4924 in Human Leukemic Cells
Source: PLoS One. 2014 Apr 1;9(4):e93530. doi: 10.1371/journal.pone.0093530 (PMC3972249; doi:10.1371/journal.pone.0093530)
Supplement: File S1 — Contains supporting information methods, tables, figures and references. (DOC) [file pone.0093530.s001.doc]

# Supporting Information File S1

## Supporting Information Methods

### Compounds and reagents

MLN4924 was provided by Dalton Medicinal Chemistry (Toronto, ON, Canada). Compound 1 was synthesized in our laboratory and prepared as described below. Both compounds were dissolved in dimethyl sulfoxide (DMSO), aliquotted, and stored at -20ºC. Where not specifically indicated, reagents were obtained from Sigma-Aldrich, Fisher Scientific, or VWR International.

### Chemical methods: Preparation of Compound 1

**a. Protection of 2’,3’-diol (compound i):** After dissolving 6-chloropurine riboside (1.0 eq) in acetone at room temperature, p-toluene sulfonic acid (10 eq, Sigma-Aldrich) was added and the reaction was stirred at room temperature for 3 hours. A saturated sodium bicarbonate solution (300 ml) was then added in 10 ml portions while the reaction was cooled in an ice bath. Remaining acetone was removed using a rotary evaporator, and the resulting aqueous solution was extracted three times with ethyl acetate, washed with brine, and dried over anhydrous sodium sulfate. Ethyl acetate was removed using a rotary evaporator, and the product was dried under high-powered vacuum for 15 minutes (yield = 90-95%).

**b.** **Nucleophilic aromatic substitution at C6 with amine (compound ii):** The protected starting material **compound i** was dissolved in DMSO (1.0 eq, Sigma-Aldrich) after which was added (S)-(+)-1-Aminoindan (2.0 eq, Sigma-Aldrich) and DIPEA (3.0 eq, Sigma-Aldrich). The reaction was sealed in a microwave vial under N_2_ and irradiated in a Biotage Initiator microwave reactor for 40 minutes at 105°C. After completion, the solution was extracted three times with ethyl acetate, washed with brine, and dried over anhydrous sodium sulfate. Ethyl acetate was removed using a rotary evaporator, and the resulting crude product was purified through a silica gel column using a Biotage Isolera One automated column device in a gradient of ethyl acetate and hexanes (yield = 85-90%).

**c. Sulfonamide formation at 5’-OH (compound iii):** A 15 ml sample of anhydrous tetrahydrofuran (THF; Sigma-Aldrich) was used to dissolve **compound ii** (1.0 eq). The solution was kept under an atmosphere of N_2_ from this moment until completion of the reaction. Following a cooling period in an ice-water bath, 1.5 eq of NaH (Sigma-Aldrich) was added to the solution, where it reacted for 30 minutes in ice-water temperature (0-4°C). Solid sulfamoyl chloride (1.5 eq, made in-house) was then added and allowed to react for a further 2 hours at room temperature. Once the reaction was complete, the solution was quenched with methanol (while cooling in an ice bath) to deactivate any remaining NaH, and was then concentrated using a rotary evaporator. The resultant product was dissolved in water and extracted three times with ethyl acetate, washed with brine, and dried over anhydrous sodium sulfate. Ethyl acetate was removed using a rotary evaporator, and the crude product using a Biotage Isolera One automated column device, in a gradient of dichloromethane and methanol (yield = 70-80%).

**d. Deprotection of 2’,3’-acetal (Compound 1) :** TFA and distilled water (3:1 ratio) were added to a round-bottom flask containing **compound iii** (1.0 eq) and the resultant solution was allowed to react for 1.5 hours at room temperature. The solution was then concentrated using a rotary evaporator, while the product residue was azeotroped three times with methanol and a further three times with chloroform, after which it was purified manually using a silica column. Solvent gradients consisted of 76% CH_2_Cl_2_, 21% methanol, and 3% ammonium hydroxide (yield = 80-85%).

### Cell culture and selection of MLN4924-resistant cell lines

K562 and U937 leukemia cell lines were maintained in RPMI 1640 media supplemented with 10% fetal bovine serum (Hyclone), 100 IU/ml penicillin G, and 100 μg/ml streptomycin. To generate MLN4924-resistant cell lines, cells were cultured in media containing stepwise increasing concentrations of MLN4924 over a 6-month period, starting from concentrations of 25 nM up to 250 nM for K562 and 5 nM up to 200 nM for U937. K562 and U937 cells that grew normally in the presence of high concentrations of MLN4924 (250 nM for K562 and 200 nM for U937) were used for further analysis.

### Cell viability assays

Cell viability was primarily determined by CellTiter-Glo assays (Promega) according to the manufacturer’s instructions. Cells (5 x 10^3^ per well) were seeded in 96-well plates and incubated with drugs. CellTiter-Glo reagents were added and plates were read using a microplate reader (Molecular Devices). Cell growth and viability were also assessed by MTS (3-(4,5-Dimethyl-thiazol-2yl)-5-(3-carboxymethoxyphenyl)-2-(4-sulfophenyl)-2H-tetrazolium; Promega) assays and trypan blue (Invitrogen) dye exclusion assays, as previously described.

### NEDD8 knockdown by lentivirus-delivered RNAi

The construction of lentiviral pLKO.1 vectors carrying a puromycin resistance gene and expressing shRNA sequences, and production of RNA viruses have been described in detail elsewhere.[^3^](#_ENREF_3) The shRNAs targeting the human NEDD8 coding sequence were as follows: 5’-GAAAGGAGATTG AGATTGACA-3’ (shNEDD8-1) and 5’- GCAAGCAGATGAATGA TGAGA-3’ (shNEDD8-2). Lentiviral infections were performed as previously described.[^3-5^](#_ENREF_3) Successfully transduced cells were selected and maintained in culture media containing 2 µg/ml puromycin.

### Immunoblot analyses

For immunoblot analyses, cells were washed with PBS and lysed in 1 x SDS sample buffer (60 mM Tris-HCl, pH 6.8, 2% SDS, 10% glycerol). Protein concentrations were determined by using the *DC* Protein Assay kit (Bio-Rad, Mississauga, ON, Canada). Subsequently, lysates were supplemented with 5% β-mercaptoethanol, heated to 95 ^o^C for 5 min, fractionated on 10% SDS-polyacrylamide gels, and transferred to nitrocellulose membranes using a gel transfer module (Criterion Blotter, Bio-Rad). Primary antibodies were detected with appropriate secondary antibodies (GE Healthcare Biosciences). Hybridization to antibodies was performed following the manufacturers’ instructions, and signals were visualized using an enhanced chemiluminescence kit (West Pico Reagent, Pierce; Rockford, IL).

### Recombinant protein production and ATP:PPi exchange assays

NAE complexes containing UBA3, UBA3 I310N, or UBA3 Y352H were purified by Ni-NTA chromatography.[^6^](#_ENREF_6) Protein concentrations were measured by quantifying Coomassie-stained gels scanned and analyzed on a LI-COR Odyssey imaging system (LI-COR Biosciences) using BSA standards. NAE, NAE (UBA3 I310N), or NAE (UBA3 Y352H) were mixed with titrants (NEDD8, ATP, PPi, and MLN4924) and other assay components, including 50 cpm/pmol [^32^P] PPi. Reactions (25 µl final volume) were initiated by the addition of NEDD8, incubated at 37ºC for 30 min, and terminated with 225 µl stop solution (5% TCA, 10 mM PPi). The NEDD8 concentration used for each enzyme (1.56 μM for NAE and 6.25 μM for both NAE (UBA3 I310N) and NAE (UBA3 Y352H)) was empirically determined from titration experiments (see Figure 4A and 6A) prior to ATP, PPi, and MLN4924 titration experiments. Quenched reactions were transferred to activated charcoal filter paper. Filters were washed in 2% TCA, 10 mM PPi, exposed to a phosphor screen, and imaged on a Fuji FLA-5100 fluorescent image analyzer (GE Healthcare, Piscataway, NJ). Resulting signals representing NAE-generated radiolabeled ATP were quantified using Multi Gauge software (GE Healthcare, Piscataway, NJ) and ATP standards.

### Statistics and data analysis

EC_50_ values were calculated using either Prism (GraphPad) or the web-based application BioDataFit (www.changbioscience.com). Data from the ATP:PPi exchange assays were analyzed using Prism software (GraphPad) and a nonlinear regression algorithm. DNA sequencing data were visualized using Chromas (Technelysium) and analyzed using BLAST (NCBI). Experiments were performed at least in triplicate and representative experiments are shown, except where indicated.

## Supporting Information Tables

***Supporting Information Table S1:* Primers used in sequencing analyses.**

| **cDNA** | **Primer type** | **Forward primer sequence** | **Reverse primer sequence** |
| --- | --- | --- | --- |
| UBA3 | RT-PCR, amplification for sequencing | 5’-GGAAGAGGCGGAGAACAAT-3’ | 5'-CGATTCAACTTCTTAGCATCCA-3' |
|  | Sequencing | 5’-GGAAGAGGCGGAGAACAAT-3’  5’-GCTTAGGATGTGAGCTCCTG-3’  5’-GTGGACTGGACTCTATCATCG-3’  5’-GGATGTTGCAGTGGCCTAAG-3’  5’-AACTGCCCAGCTTGTAGCCAG-3’ | 5’-CGATTCAACTTCTTAGCATCCA-3’  5’-CTGGCTACAAGCTGGGCAGTT-3’  5’-CTTAGGCCACTGCAACATCC-3’  5’-CGATGATAGAGTCCAGTCCAC-3’  5’-CAGGAGCTCACATCCTAAGC-3’ |
| NEDD8 | RT-PCR, amplification for sequencing | 5’-GAGCGGTAGGAGCAGCAAT-3’ | 5’-GGCTATGGTGTCCCAGAGAG-3 |
|  | Sequencing | 5’-GAGCGGTAGGAGCAGCAAT-3’ | 5’-GGCTATGGTGTCCCAGAGAG-3 |

***Supporting Information Table S2 (related to Figure 6A):* Growth inhibition (EC_50_) of parental K562 and MLN4924-resistant R-K562MLN cells by selective NAE inhibitors.**

| **Compound** | **EC_50_ in K562 (µM)^*^** | **EC_50_ in R-K562_MLN_ (µM)** |
| --- | --- | --- |
| 49 | 380 ± 223 | > 1000 |
| 14 | 132 ± 99 | > 1000 |
| 22 | 203 ± 24 | > 1000 |
| 13 | 160 ± 88 | > 1000 |

^*^EC_50_ values for compounds in parental K562 cells have been previously reported by our group in Lukkarila et al.[^7^](#_ENREF_7)

## Supporting Information Figures


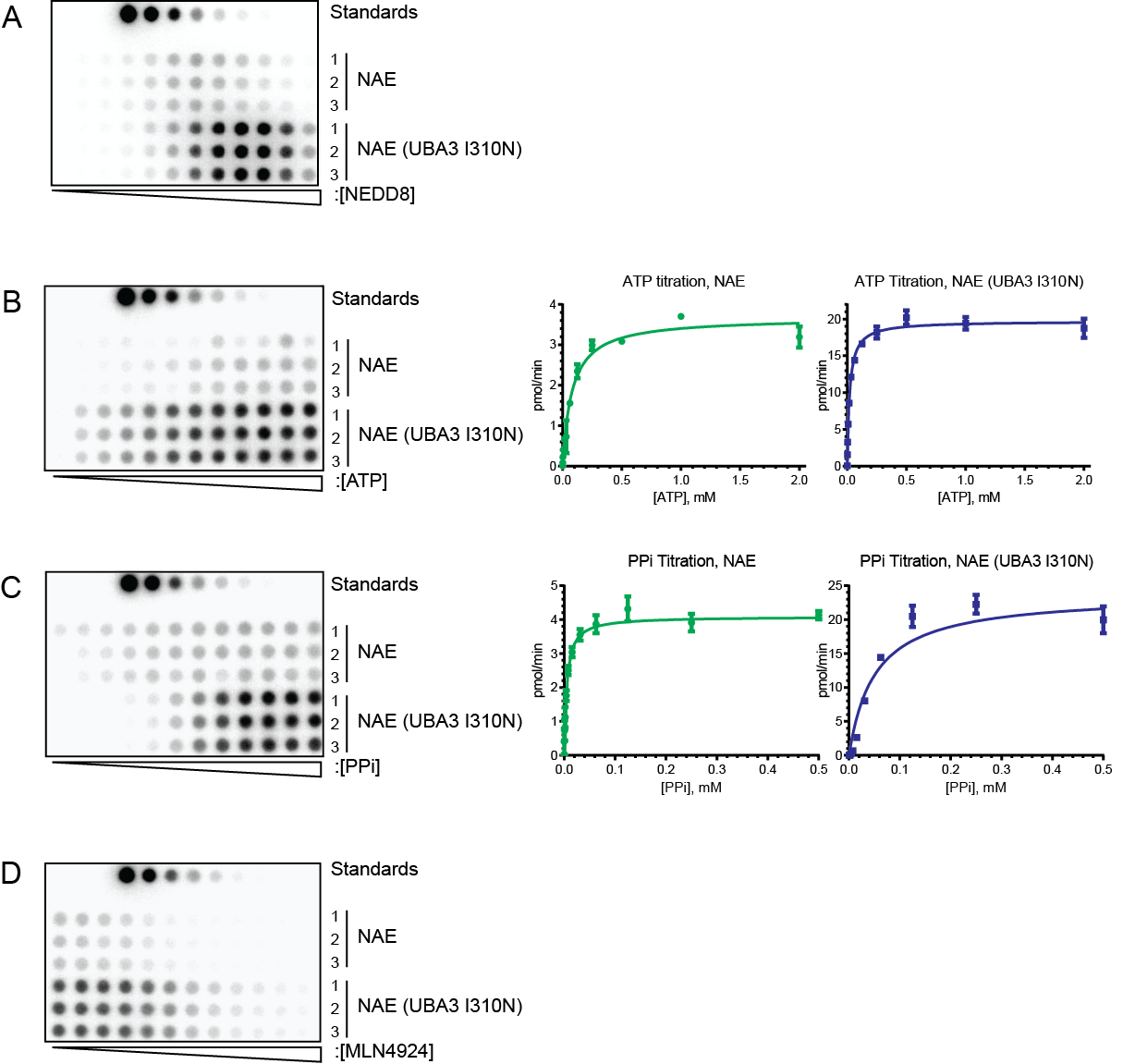


***Supporting Information Figure S1 (related to Figure 3):* Raw data from ATP:PPi exchange assays.** Phosphor screen scans of ATP synthesis by NAE and NAE containing the UBA3 I310N mutation in titrations with NEDD8 **(A)**, ATP **(B)**, PPi **(C)**, and MLN4924 **(D)**. Experiments were performed in triplicate and the ATP synthesized was quantified using radiolabeled ATP standards. Titrations were performed with either 2-fold (NEDD8, ATP, and PPi) or 3-fold (MLN4924) serial dilutions with other reaction components in excess, except for NEDD8, which was used at an empirically-determined concentration for each NAE complex that gave the maximum rate of ATP synthesis. Error bars indicate SEM.


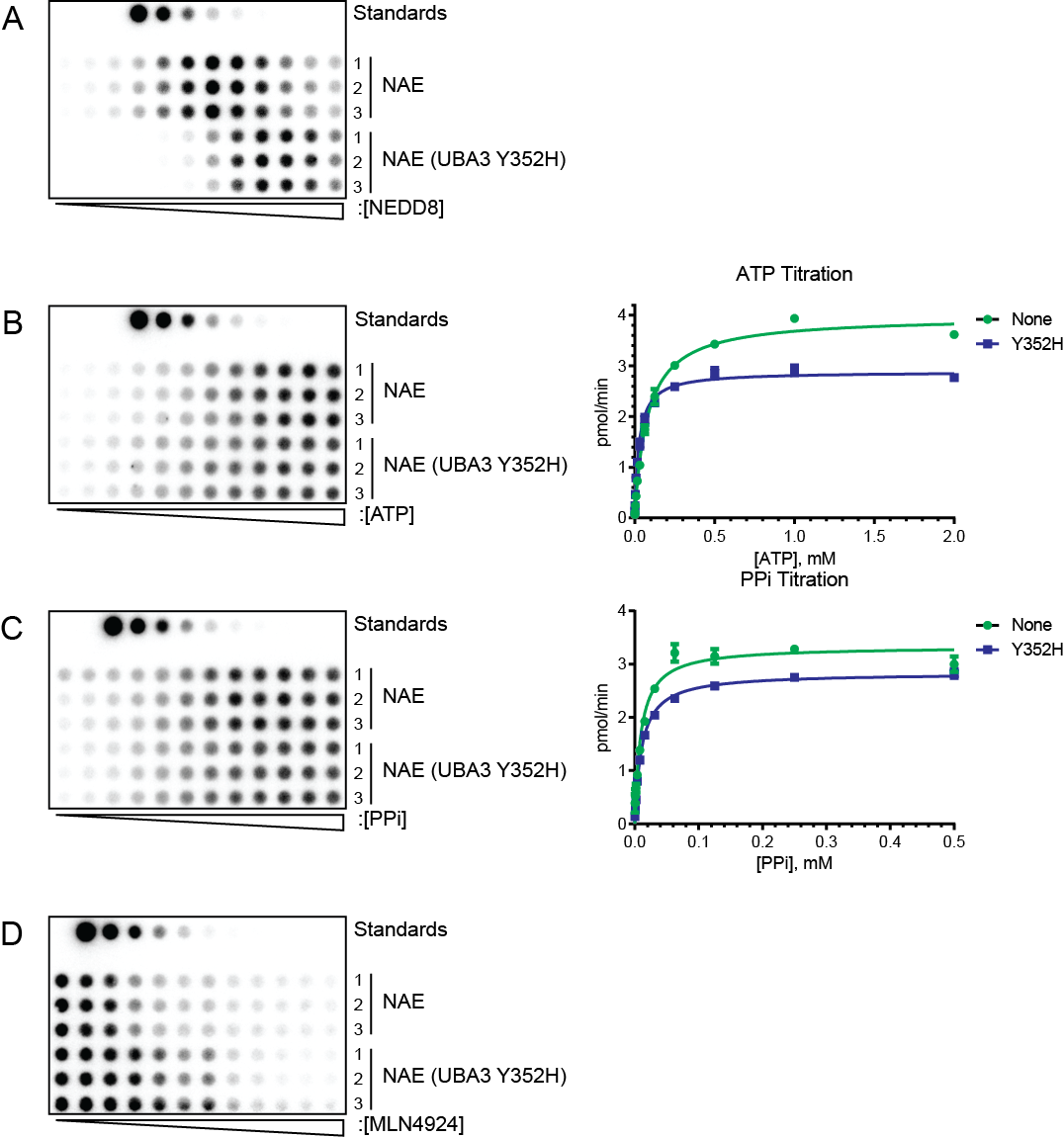


***Supporting Information Figure S2 (related to Figure 5):* Raw data from ATP:PPi exchange assays.** NAE and NAE (UBA3 Y352H) were evaluated in ATP:PPi exchange assays, titrating NEDD8 **(A)**, ATP **(B)**, PPi **(C)**, or MLN4924 **(D)**. Shown are phosphor screen scans of the filters used to measure rates of ATP synthesis with respect to the indicated titrants and as shown in Figure 7. Error bars indicate SEM.

## Supporting Information References

1. Carter BZ, Gronda M, Wang Z, et al. Small-molecule XIAP inhibitors derepress downstream effector caspases and induce apoptosis of acute myeloid leukemia cells. *Blood.* 2005;105(10):4043-4050.

2. Schimmer AD, Thomas MP, Hurren R, et al. Identification of small molecules that sensitize resistant tumor cells to tumor necrosis factor-family death receptors. *Cancer Res.* 2006;66(4):2367-2375.

3. Moffat J, Grueneberg DA, Yang X, et al. A lentiviral RNAi library for human and mouse genes applied to an arrayed viral high-content screen. *Cell.* 2006;124(6):1283-1298.

4. Skrtic M, Sriskanthadevan S, Jhas B, et al. Inhibition of mitochondrial translation as a therapeutic strategy for human acute myeloid leukemia. *Cancer Cell.* 2011;20(5):674-688.

5. Xu GW, Ali M, Wood TE, et al. The ubiquitin-activating enzyme E1 as a therapeutic target for the treatment of leukemia and multiple myeloma. *Blood.* 2010;115(11):2251-2259.

6. Toth JI, Yang L, Dahl R, Petroski MD. A gatekeeper residue for NEDD8-activating enzyme inhibition by MLN4924. *Cell Rep.* 2012;1(4):309-316.

7. Lukkarila JL, da Silva SR, Ali M, et al. Identification of NAE inhibitors exhibiting potent activity in leukemia cells: exploring the structural determinants of NAE specificity. *ACS Med Chem Lett.* 2011;2(8):577-582.
